# Image-Guided Transcranial Focused Ultrasound Stimulates Human Primary Somatosensory Cortex

**DOI:** 10.1038/srep08743

**Published:** 2015-03-04

**Authors:** Wonhye Lee, Hyungmin Kim, Yujin Jung, In-Uk Song, Yong An Chung, Seung-Schik Yoo

**Affiliations:** 1 Incheon St. Mary's Hospital, The Catholic University of Korea, Incheon 403-720, Korea; 2 Center for Bionics, Korea Institute of Science and Technology, Seoul 136-791, Korea; 3 School of Nano-Bioscience and Chemical Engineering, Ulsan National Institute of Science and Technology, Ulsan 689-798, Korea

## Abstract

Focused ultrasound (FUS) has recently been investigated as a new mode of non-invasive brain stimulation, which offers exquisite spatial resolution and depth control. We report on the elicitation of explicit somatosensory sensations as well as accompanying evoked electroencephalographic (EEG) potentials induced by FUS stimulation of the human somatosensory cortex. As guided by individual-specific neuroimage data, FUS was transcranially delivered to the hand somatosensory cortex among healthy volunteers. The sonication elicited transient tactile sensations on the hand area contralateral to the sonicated hemisphere, with anatomical specificity of up to a finger, while EEG recordings revealed the elicitation of sonication-specific evoked potentials. Retrospective numerical simulation of the acoustic propagation through the skull showed that a threshold of acoustic intensity may exist for successful cortical stimulation. The neurological and neuroradiological assessment before and after the sonication, along with strict safety considerations through the individual-specific estimation of effective acoustic intensity *in situ* and thermal effects, showed promising initial safety profile; however, equal/more rigorous precautionary procedures are advised for future studies. The transient and localized stimulation of the brain using image-guided transcranial FUS may serve as a novel tool for the non-invasive assessment and modification of region-specific brain function.

Development of novel brain stimulation modalities would offer new opportunities in the creation of functional mapping probes for basic neuroscientific research as well as tools for non-pharmacological neurotherapeutic interventions^1^,^2^. Invasive approaches such as deep brain stimulation (DBS) or epidural cortical stimulation (EpCS)^2^ have a limited scope of application due to the accompanying surgical procedures. Non-invasive brain stimulation methods, such as transcranial magnetic stimulation (TMS) or transcranial direct current stimulation (tDCS), modulate the function of cortical areas without surgery^1^,^3^. However, they lack spatial specificity and depth penetration^2^,^4^ and, therefore, warrant the advent of a more localized means of brain stimulation with the capability of reaching deep brain tissues. Recently, the optogenetic approach has been proposed as a brain stimulation method with the ability to control the activity of an individual/group of neural cells in the brain^5^,^6^, yet the genetic modification required for the induction of light-sensitive neurons, along with limited penetration of the stimulatory light, may hinder its immediate application in humans.

The use of ultrasound has been suggested for the functional modulation of the central nervous system (detailed review can be found in Bystritsky *et al.*^7^). Since the seminal investigation by Fry *et al.*, whereby ultrasound sonication of the lateral geniculate nucleus resulted in reversible inhibition of the visual evoked potential (VEP) in cats^8^, the neuromodulatory potential of ultrasound has been demonstrated *via* experimentation on both *ex vivo*^9^,^10^ and *in vivo* rodent brains^11^. In humans, auditory sensations^12^ and mood changes among chronic pain patients^13^ have been associated with the administration of transcranial Doppler ultrasound, suggesting that ultrasound may have an impact on modulating the function of the brain. The spatial specificity and depth penetration of the ultrasound stimulation are provided by the use of the focused ultrasound (FUS) technique, whereby highly-focused (having a focal size measuring a few millimeters) acoustic energy is delivered to the biological tissue through the use of the geometric shape of the transducer^14^,^15^, the acoustic lens^16^, or the phased actuation of multiple FUS elements^17^,^18^. Challenges in transcranial application of FUS to the brain, such as sound absorption and refraction by the skull, have been overcome by adopting a phased FUS transducer array^17^ operating at a low fundamental frequency (typically < 1 MHz, whereby a much higher range of 1–15 MHz is used for clinical imagers). Subsequently, transcranial high intensity focused ultrasound (HIFU) has been utilized for functional neurosurgery through tissue ablation^19^,^20^ under on-line image-guidance using magnetic resonance imaging (MRI) for accurate anatomical targeting.

By employing a low acoustic energy that is far below the level that induces temperature changes in the brain tissue, FUS has recently been investigated as a new mode of region-specific brain stimulation. The ability to reversibly modulate region-specific excitability of the sonicated brain tissue has been demonstrated by the excitation or suppression of neural activity *via* sonication of the motor and visual areas in rabbits^21^, the suppression of chemically-induced epilepsy^22^, and the alteration of extracellular concentrations of neurotransmitters in rats^23^,^24^. In non-human primates, FUS given to the frontal eye field of macaques modulated their visuomotor behavior^25^. Recent investigation by Legon *et al*.^26^ showed that the amplitude of sensory-evoked electroencephalogram (EEG) potentials was modulated *via* FUS sonication on the primary somatosensory cortex (S1) in humans along with enhanced behavioral performance in the two-point tactile discrimination tasks. Yet, the question remains as to whether the FUS-mediated stimulation of the S1 in the brain could elicit explicit somatosensory sensations in the absence of external sensory stimulation.

We were motivated to transcranially apply low-intensity FUS to the human hand S1 of the brain, and we subsequently examined the presence of elicited sensory responses on the fingers and hand, including the type and location of responses. To quantitatively examine the influence of FUS stimulation, we also measured the cortical EEG potentials evoked by the FUS. Due to the individual variations in cranial structures as well as anatomical and functional neuroanatomy, multi-modal image-guidance using computerized tomography (CT) and magnetic resonance imaging (MRI) was employed to target the sonication focus to the desired brain area.

## Results

### Location and Type of Sensations elicited by FUS Stimulation

**Figure 1:**
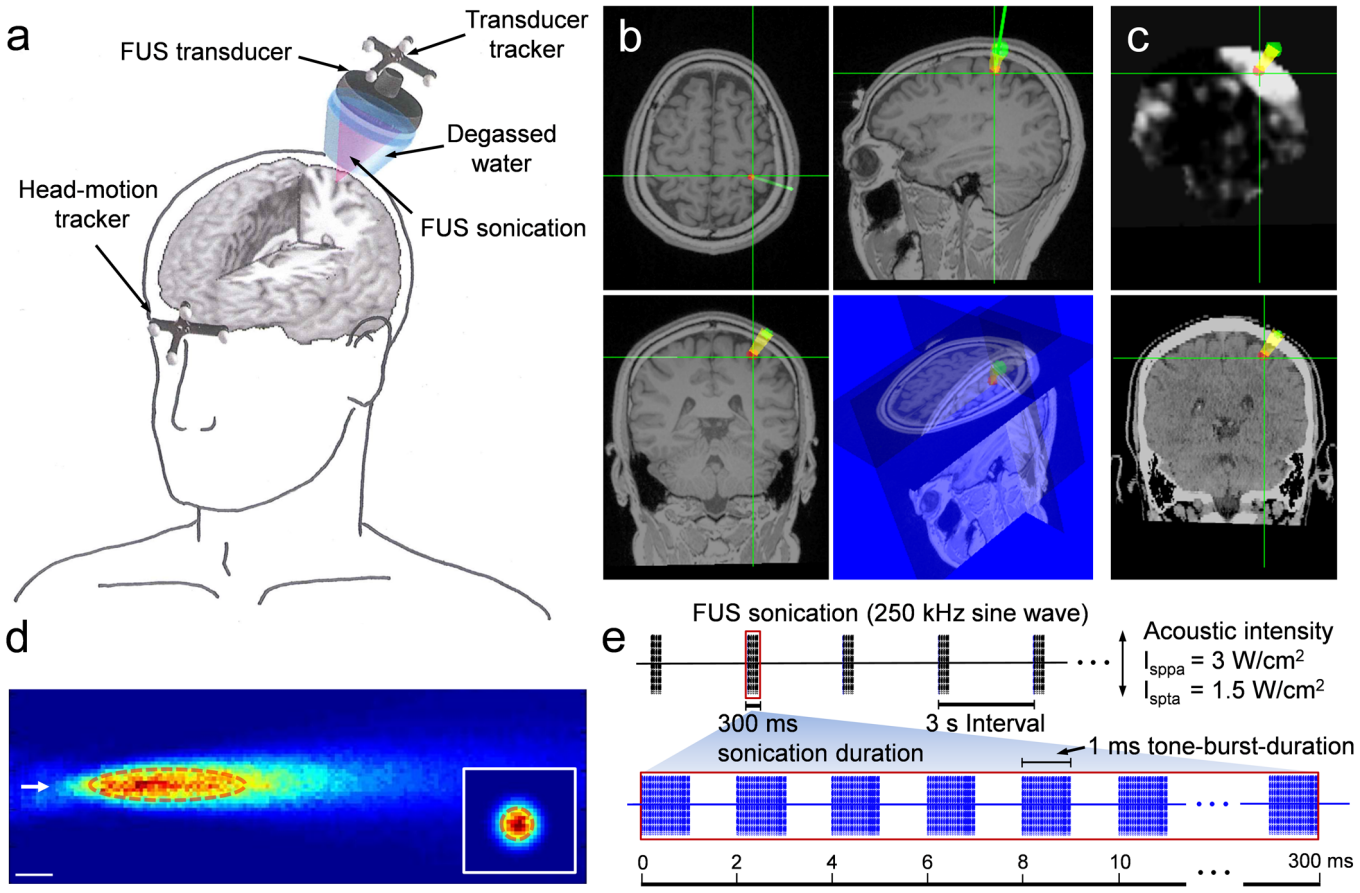
Schematics of the image-guided FUS sonication setup and parameters. (a) The acoustic focus of the FUS transducer was positioned on the targeted brain area using the spatial information (coordinates and orientations in space) provided by the optical trackers that are attached to the FUS transducer and the forehead. Each tracker contained four infrared-reflective markers to be detected by a motion tracking camera. (b) Representations of the planned target (the red dot) and path (in yellow) of the sonication overlaid on the anatomical MRI. The entry point on the scalp (green circle) on the left somatosensory cortex and real-time display of the focal location (green crosshairs) and the path of the sonication (angled green line) are also shown. (c) The functional MRI (*t*-value map, upper panel) and cranial CT (lower panel) data on the same location, which can be selected by the operator to monitor the location of the focus and incident angle of the sonication path relative to the skull. (d) The spatial profile of the acoustic intensity at the sonication focus generated by the 250 kHz FUS transducer in the longitudinal (50 × 150 mm^2^ rectangular area, 1 mm step) and the transversal (inset, 30 × 30 mm^2^ square area, 1 mm step) planes of the sonication. The arrow indicates the direction of the sonication. A cigar-shaped (47 mm in length and 7 mm in diameter) acoustic focus based on the full-width at half-maximum (FWHM) of the acoustic intensity is depicted by the dashed line. Scale bar, 10 mm. (e) The schematics of the acoustic parameters used to generate the pulsed FUS for the transcranial brain stimulation to elicit the excitation in the somatosensory cortex. The batches of sinusoidal acoustic pressure waves at 250 kHz, each 300 ms in sonication duration, were given every 3 s. Each batch consisted of a 1 ms burst of sonication pulses (*i.e.*, tone-burst-duration) operating at a pulse repetition frequency of 500 Hz (*i.e.* duty cycle of 50%). This figure was drawn by W.L., H.K. and S.-S.Y.

We used individual-specific MRI data, including both anatomical and functional information, as well as cranial CT data to guide the transcranial application of the low-intensity FUS sonication to the human hand S1 of the brain (Fig. 1). 11 participants (out of a total of 12 subjects) reported various types of sensations (subject ‘h5' reported no sensations) that occurred mostly at the hand area contralateral to the sonicated hemisphere (Table 1 for subject-specific occurrences). Peripheral sensations of the scalp, similar to those experienced during the application of repetitive TMS (rTMS)^27^,^28^,^29^, were not present.

The locations of the tactile sensations (without considering their types) were tabulated in Table 1a. All FUS-responsive subjects (*n* = 11) reported the elicited sensations on the hand area and/or finger(s) contralateral to the side of sonication. Sensations were reported in 45% of the subjects (*n* = 5) on the forearm and in 36% of the subjects (*n* = 4) over the entire arm, including the upper arm (excluding the shoulder). Locations such as the wrist (*n* = 2), elbow (*n* = 2), and armpit (*n* = 1) were also reported. Three of the subjects (‘h2', ‘h7', and ‘h8') reported that the sensations started from the forearm or wrist and ‘radiated' towards either the elbow or finger(s). While all these locations were limited to the upper limb area, there was one occasion (from ‘h6') whereby the sensation was also felt in the buttock and foot areas (also on the contralateral side of sonication).

**Figure 2:**
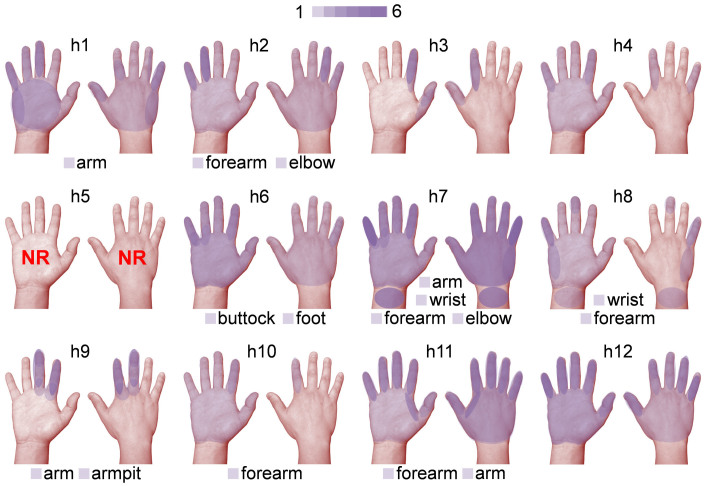
Illustration depicting the locations of tactile sensations experienced by the subjects under FUS stimulation. The regions of sensations felt from the left and right hands, including the wrist, as represented by the semi-transparent purple layers, were merged onto the palmar (left) and dorsal (right) view of the right hand (‘h1' through ‘h12'). The number of occurrences for a set of distinctive locations of sensation are represented by a color scale (1–6). The locations of other reported sensations (*i.e.* arm, forearm, armpit, elbow, wrist, buttock, and foot) were labeled at the bottom of each panel. Subject ‘h5' did not report any sensations (noted as ‘NR').

The locations of the sensations in the hand area, in terms of the number of occurrences as a distinctive response event, are illustrated in pseudo-color on the palmar and dorsal hand for each subject (Fig. 2; the regions of sensations felt from both hands, including the wrist, were merged together on the right hand). The sensations were felt around the hand and/or the finger(s) area, sometimes differentially on either the palmar or the dorsal side of the hand. Among the fingers, the sensations were felt from a single finger or from a group of two to three neighboring fingers, especially following the innervation patterns of distinctive hand nerve groups (*i.e.* radial, median, and ulnar). We did not, however, observe sensations elicited in non-adjacent fingers.

Ten subjects reported more than one type of sensation, occurring either as independent events or mixed with others (described in Table 1b). All responsive individuals reported ‘tingling' as well as ‘feeling a part of the hand moving or twitching', termed as sensation of movement (SOM)^30^,^31^. About half of the individuals (*n* = 6) reported feeling of ‘heaviness' while others experienced ‘numbness' (18.2%, *n* = 2), a ‘feeling of weak electrical current flow' (27.3%, *n* = 3), and ‘itching' (18.2%, *n* = 2). One of the subjects also reported feelings of ‘brushing' and ‘cooling.' Regardless of the type, these sensations were distinctively synchronized with the sonication trials that were given 3 s apart, and phased out before the onset of the next sonication trial. The sham-type stimulation condition (given without actual sonication, but otherwise mimicking the same environment of the sonication) did not elicit any sensations across the subjects.

Not all of the FUS stimulation trials resulted in the elicitation of a response from the subjects (Supplementary Table S3, the percentage of the FUS stimulations that elicited responses for each subject's left and right hemispheres, respectively). There was a degree of variability in the response rate among the individuals, ranging from 88–89% in one subject (‘h2') to a marginal 12–15% in another subject (‘h10'). On average, approximately half of the sonication trials (54.4 ± 23.5%, mean ± s.d.; *n* = 24) were found to elicit responses.

### Electroencephalographic Cortical Potential evoked by FUS Stimulation

**Figure 3:**
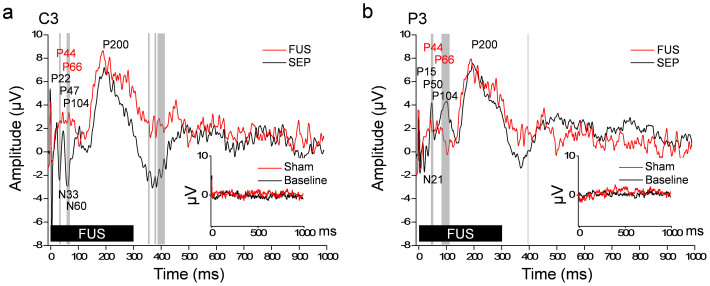
Electroencephalographic (EEG) evoked potential elicited by image-guided transcranial FUS to the hand primary somatosensory cortex. The grand average (*n* = 6, the subjects ‘h13' through ‘h18') evoked potentials of the EEG electrode sites C3 (a) and P3 (b) were shown in the case of median nerve stimulation (100 trials, SEP, black line) and transcranial FUS stimulation (100 trials, red line, noted as ‘FUS'). The FUS was given at the initial period of 300 ms (thick solid black bar). Positive (noted with prefix P) and negative (noted with prefix N) peaks of the SEP are annotated across the two electrode sites. Gray vertical bars indicate the time-segments that showed significant differences (paired *t*-test, two-tailed, *P* < 0.05) in amplitudes between the SEP and the FUS-mediated evoked potentials. The inset shows the EEG signals measured at the same electrode sites from the baseline (*i.e.* no stimulation) and sham FUS conditions.

The participants who underwent EEG recordings during FUS stimulation of the S1 also reported sensations such as ‘tingling' and SOM on the finger(s), hand, and arm areas contralateral to the stimulated hemisphere. In the review of somatosensory evoked potentials elicited by median nerve stimulation (SEP, Fig. 3, grand averaged *n* = 6), classically-defined EP components were detected, for example, P22, N33, P47, N60, P104, and P200 (late potential; LP) at C3 site (Fig. 3a), and P15, N21, P50, P104, and LP at P3 site (Fig. 3b). The FUS-mediated EP shared similar features with those of the SEP in the mid-latency (P45 and P66, although relatively small in amplitude) as well as in the long-latency time domains (*i.e.* P200). They were, however, different (*via* paired *t*-test, two-tailed, *P* < 0.05) among a few time-segments in the mid- (including N33/N60 from C3 site and P50 from P3 site) and long-latency time domains (>100 ms post-stimulation^32^, *i.e.* 350 and 380 ms at C3 site, and P104 and 400 ms at both C3 and P3 sites). The difference was also noted in the short-latency domain (<25 ms post-stimulation^32^) that did not show any distinct peaks in the FUS-mediated EP. EEG measured during the sham FUS stimulation sessions (Fig. 3, insets, noted as ‘Sham') did not generate any distinct peaks, which was similar to the ones acquired without any external stimuli (Fig. 3, insets, noted as ‘Baseline'). Among the participations, two reported transient (only present in few stimulation episodes) ‘tingling' sensations at the scalp (at the sonication path) during the middle of the FUS session. This scalp sensation was not likely to affect the time-averaged evoked potential (EP) components due to its transient nature.

### Acoustic Simulation of Transcranial FUS across the Individuals

**Figure 4:**
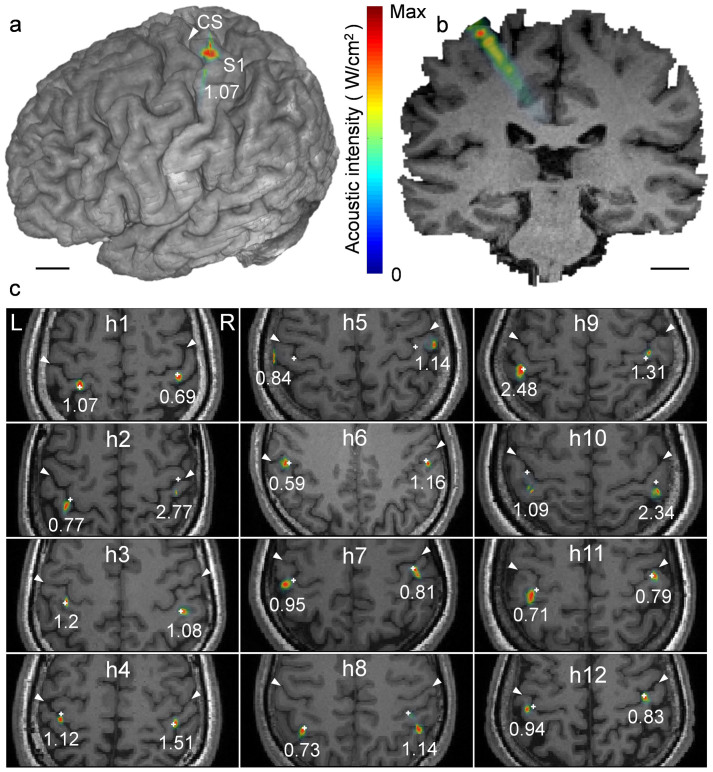
Simulated acoustic intensity profiles overlaid on the region of the hand primary somatosensory cortex. The acoustic intensity profiles projected on (a) a 3D rendering and (b) a coronal section of the volumetric MRI data (from ‘h1'). The value, 1.07, indicates the maximum I_sppa_ value (in W/cm^2^) at the focus. Scale bar, 1 cm. (c) The simulation results displayed on the axial view of each individual's anatomical MRI (‘h1' through ‘h12'; white arrows indicate the subject's central sulci; CS). The location of the intended FUS focus is marked with ‘+'. The numbers under each simulated focus indicate the maximum acoustic intensities (I_sppa_ in W/cm^2^).

The simulated acoustic intensity profile (pseudo-colored and overlaid on the anatomical MR images; Fig. 4) showed that the acoustic focus was successfully projected on the postcentral gyrus (*i.e.,* the S1). The profile of the simulated acoustic intensity longitudinal to the sonication beam path (Figs. 4a and b, the case of subject ‘h1') indicates that the acoustic energy was focused at the targeted cortex. The simulated acoustic intensity profiles overlaid on the anatomical MRI for individual subjects from ‘h1' to ‘h12' (Fig. 4c) revealed that the FUS focus was positioned to the targeted somatosensory cortex (marked with a white ‘+') within a few millimeters for most of the subjects. However, rather large deviations of greater than 10 mm were observed in the case of ‘h5' and ‘h10'.

The acoustic intensity at the intended target (AI_@target_) and its maximum value within its surroundings (AI^max^_@ROI_), as well as the estimated spatial deviation of the FUS focus from the intended target, were tabulated in Table 2. The skull thickness measured along the sonication path was also shown. The estimated acoustic intensity at the intended target had a spatial-peak pulse-average acoustic intensity (I_sppa_) of 0.7 ± 0.5 W/cm^2^ (mean ± s.d., *n* = 24), which was only about 24% of the incident I_sppa_ of 3 W/cm^2^, suggesting that 76% of the incident intensity, on average, was attenuated during the transcranial application of the FUS. It is noteworthy that simulation from ‘h5' (who was the only one who did not report any sensation) showed the largest amount of spatial deviation from the intended target (a deviation of greater than 10 mm) and one of the lowest estimated acoustic intensity (I_sppa_ of 0.2 W/cm^2^) among all of the participants. The same subject happened to have the greatest skull thickness (*i.e.* >10 mm). Of all the subjects who participated in the EEG study (*n* = 6, named ‘h13' through ‘h18'), the targeting accuracy remained less than the diameter of the focus (the focus shifting = 0.9 ± 1.2 mm; mean ± s.d.). The estimated acoustic intensity at the intended target was 1.0 ± 0.4 W/cm^2^ I_sppa_ (mean ± s.d.; detailed information can be found in the Supplementary Table S4).

### Post-Sonication Follow-Up and Neurological and Radiological Assessment

Neurological exam (NE) conducted by physicians who were blinded to the nature of study did not reveal any abnormal findings before and after the sonication across all subjects. The follow-up anatomical MRI readings, conducted at four different time periods, immediately (*n* = 7), 2 weeks (*n* = 3), 4 weeks (*n* = 6), and 8 weeks (*n* = 2) after sonication, did not identify any radiological abnormalities. Based on the follow-up interviews (occurring 8 weeks after the sonication, 55.9 ± 0.4 days; mean ± s.d., *n* = 18), no changes in mental or physical status or discomforts associated with the procedure were reported.

## Discussion

Image-guided transcranial application of low-intensity FUS sonication to the S1 elicited not only explicit tactile sensations, but also cortical evoked potentials similar to the classical SEP generated by median nerve stimulation. Our data provide the first evidence of active creation of stimulatory responses from the brain elicited by FUS in the absence of any external tactile stimulation. The stimulatory effects were transient and reversible and did not cause any discomfort or adverse effects across the study participants.

The tactile sensations elicited by the FUS stimulation occurred in the hand area contralateral to the sonicated hemisphere. It is noteworthy that some of these sensations were felt from a finger or neighboring fingers (as shown in Fig. 2) that follow the innervation patterns of specific major nerves in the hand. Based on the presence of a spatially-distinct somatotopic arrangement of fingers within the primary somatosensory cortex, having a distance of 7–18 mm (thumb to little finger)^33^,^34^,^35^, we conjecture that transcranial FUS, having a sufficiently small acoustic focal dimension, is capable of stimulating not only the hand area of the brain but also its sub-region(s), and subsequently, elicits sensation from even a single finger.

On the other hand, the sensations were also elicited from non-hand areas (contralateral to the sonicated hemisphere) in a few individuals, including the wrist, forearm, elbow, armpit and entire arm. While most of these sensations were localized to the hand and upper limb area, one subject (‘h6') felt sensations in the buttock and foot area (in addition to the hand). We hypothesize that these sensations were associated with the misalignment of the FUS focus, which stimulated the adjacent non-hand somatosensory areas. The refracted sonication path at the skull interface, the individual variations in local neuroanatomy (for example, cortical folding), as well as the subjects' head motion during FUS trials might have contributed to the cause of the misalignment. Regarding the sensations that radiated from one part of the arm to another, for example, from the forearm to the elbow (in ‘h2'), simultaneous stimulation of neighboring cortices in the functional representation of these areas may also have been involved. The reverberation of the acoustic waves, which could occur inside the rodent skull cavity^36^, may also induce the stimulation of multiple locations of the neural tissues outside of the target; however, is not likely to be a contributing factor in the present study due to the much larger size of the human cranial structure.

Regarding the types of sensations elicited by FUS stimulation, ‘tingling' and SOM sensations were most prevalent (*n* = 11), although other sensations such as ‘heaviness', ‘numbness', ‘feeling of weak electrical current flow', ‘itching', ‘brushing' or ‘cooling' were also reported. These findings bear similarities with previous studies of direct electrical stimulation of the somatosensory cortex, whereby various tactile sensations were reported^37^,^38^. The SOM experienced by the subjects might have been associated with the coincidental stimulation of the motor circuitry (occurs in the absence of actual efferent motor output), which is often seen during TMS^30^,^31^,^39^. Further studies involving the sonication of primary motor area (M1) and its surroundings, such as pre- and supplementary motor areas, will provide more information on the probable cause for the elicitation of SOM.

In addition to subjective reporting of tactile sensations, EEG showed the distinctive peaks evoked by the FUS stimulation. The overall features of the FUS-mediated EP, including the mid- and long-latency components, were similar to those of the classical SEP generated by median nerve stimulation. The short- and/or mid-latency components of the SEP are known to be associated with the transmission of the afferent tactile signal and its subsequent processing in the S1^40^,^41^ while the long-latency components are related to the associative processing of information that occurs in the adjacent cortices^42^. We conjecture that the differences in the feature among the short-latency components (within 25 ms after stimulation), especially the absence of any distinct peaks from the FUS-mediated EP, are due to the absence of any afferent neural signal (no external stimulation), while the rest of the peaks, including the strikingly similar P200 components, reflect the successful activation of the primary sensory cortex and subsequent recruitment of neural substrates by the FUS stimulation.

Through numerical simulation of acoustic intensity profiles, we learned that most of the actual FUS focus was closely aligned with the intended target (deviation of 3.5 ± 4.1 mm; mean ± s.d.), but different levels of acoustic intensities were delivered to each sonication target. For one subject (‘h5') who failed to respond to the FUS stimulation, the acoustic intensity at the intended target was markedly small, having an I_sppa_ level of 0.1–0.2 W/cm^2^. The individual's relatively thick skull, combined with the large deviation (on the order of a centimeter) of the sonication focus from the S1, was a probable factor for this diminished acoustic intensity. These data suggest the potential existence of a threshold of acoustic intensity for successful stimulation. Similar intensity-dependent variations in the success rate have been demonstrated in the rodent model of sonication-mediated motor stimulation^43^ and in the rabbit model^21^. The use of 4 W/cm^2^ I_sppa_ in non-human primates^25^ and 23.87 W/cm^2^ I_sppa_ for behavioral modulation in humans^26^ were much higher than the that used in the present study (3 W/cm^2^ I_sppa_) and suggests the importance of deploying a sufficient level of acoustic intensity for successful FUS-mediated brain stimulation.

The presence of an intensity threshold for successful stimulation may also explain the reason why the FUS trials did not always result in the elicitation of responses, whereby approximately only half (54.4 ± 23.5%; mean ± s.d., *n* = 24) of the delivered stimulations were able to elicit sensations. We hypothesize that the inadvertent head movements and associated aberration of focus during the FUS session can cause a steep decrease in acoustic intensity at the intended target, and may result in a sub-threshold stimulatory condition. Since sonication accuracy would be largely dependent upon the method of sonication delivery/guidance and transducer geometry (and therefore the focus size/shape), further investigation is needed to increase the spatial accuracy in delivering sonication to the specific target even in the presence of potential head movements. The possible misalignment of the sonication target due to head motion can be improved by adopting a helmet-like gear that houses a FUS transducer and moves along with the head motion. The previous study by Legon *et al.* partially adopted this strategy by placing the FUS transducer based on the EEG montage sites^26^; however, this may introduce significant spatial error in positioning of the acoustic focus due to individual differences in structural and functional neuroanatomy. The use of an array-type beam steering under image-guidance^44^,^45^, adopted in commercial FUS systems, could also be considered over the single-element transducer to increase the targeting accuracy of the FUS focus.

The pulsing parameters (*i.e.* tone-burst-duration of 1 ms; pulse repetition frequency, PRF of 500 Hz; sonication duration of 300 ms) used in the present study were based on our previous animal research data and appear to have been effective in stimulating the human brain, regardless of differences in several important experimental designs. The 250 kHz fundamental frequency, lower than the frequencies used in most of our previous FUS studies^22^,^46^,^47^, was chosen to help alleviate the concerns for acoustic energy absorption or refraction by the skull. The acoustic focus was formed proximal to the targeted cortical area across the majority of the subjects, validating the choice of frequency and the use of the single-transducer setup with image-guidance to deliver the sonication to the intended target. However, the differences in individual skull thickness and variations in sonication path with respect to the skull's surface altered the attenuation and propagation of acoustic waves through the skull, consequently affecting the location and acoustic intensity of the FUS stimulation. Therefore, it is also desirable to have an on-site estimation and feedback system of the acoustic propagation, for example, by implementing a computationally-efficient acoustic simulation method^48^,^49^, which enables the on-demand adjustment of the transducer's orientation and power output for accurate targeting and delivery of acoustic energy.

As to the fundamental mechanism in explaining the FUS-mediated brain stimulation, several hypotheses are considered. Thermal contribution, which has been seen in the modulation of the peripheral nerve^50^, was excluded as a candidate mechanism due to the use of an extremely low acoustic energy for a short duration, far below the level that can heat the sonicated brain tissue (<0.01°C, see the *Estimation of Thermal Effects from the Sonication*, Methods), as also supported by our previous findings from rodents^46^ in which similar sonication parameters (but with a significantly greater number of stimulation trials per FUS session) were not likely to alter the tissue temperature. Instead of the thermal contribution, changes in transmembrane capacitance and subsequent generation of the action potential due to the exposure to the acoustic pressure waves^51^,^52^,^53^ may serve as a probable contributing factor for underlying stimulatory effects. Mechanical activation of glial cells *via* mechanoreceptors^54^ may also serve as an underlying mechanism. Further studies, probing the *in vitro*/*in vivo* cellular responses to sonication, are urgently needed to elucidate the detailed mechanism of ultrasound-mediated brain stimulation.

Based on neurological and neuroradiological assessment, the sonication parameters used in this study appear to be safe, with an acoustic intensity of 2.5 W/cm^2^ I_sppa_ (maximum simulated value observed in the left hemispheric sonication in ‘h9'). The corresponding mechanical index (MI) was 0.62, which is much lower than the American Food and Drug Administration (FDA) safety guideline limit of 1.9 for soft tissue sonication^55^. The estimated spatial-peak temporal-average acoustic intensity (I_spta_) at the target, averaged across the responsive subjects, was on the order of 350 mW/cm^2^ (based on the 50% duty cycle of 0.7 ± 0.5 W/cm^2^ I_sppa_; Table 2), which was also lower than the maximum allowed sonication intensity of 720 mW/cm^2^ I_spta_ by the FDA in ultrasound imagers^55^. These results are in good agreement with current safety records in animal studies, and they cast a promising possibility for the safe administration of neuromodulatory FUS for use in humans.

The ability to non-invasively stimulate a region-specific area of the human brain, supported by the evidence of transient suppression in brain activity in animal studies^21^,^22^, may lead to new breeds of applications in the field of neuroscience and clinical medicine. For example, the FUS-mediated functional modulation of a specific brain area may also be used as a new method for non-invasive functional brain mapping. Based on the evidence of its ability to alter the extracellular level of neurotransmitters in the rodent model^23^,^24^, FUS may shed light on providing new modes of neurotherapeutics for the treatment of neurotransmitter-mediated psychiatric disorders. Furthermore, when combined conjunctively with FUS functional neurosurgery techniques^19^,^20^, the presented method may be used to evaluate the function of the targeted surgical area prior to its thermal ablation. Although the present method was applied on the cortical area, the ability to deliver the acoustic energy to a deep brain structure may be used to selectively modulate important functional areas, such as, but not limited to, the hippocampus or thalamus, and may confer a new window of opportunities for treating neurological disorders. To fully realize these potential capabilities of FUS stimulation in the human brain, systemic exploration and optimization of various combinations of acoustic parameters for effective and safe sonication are needed, including the investigation of repeatability and safety of the method through multi-session administration of FUS stimulation.

## Methods

### Overview of Experimental Procedures

Healthy human volunteers (*n* = 18, five females, age 29.2 ± 6.4 yrs, range 21–43) participated in this study under the approval of the Institutional Review Board (IRB; Incheon St. Mary's Hospital, The Catholic University of Korea). The methods were carried out in accordance with the approved guidelines. All participants gave written consent prior to participation. Enrolled subjects did not have any history of central or peripheral nerve diseases. 12 individuals (four females, age 29.4 ± 5.0 yrs, range 25–41, named from ‘h1' through ‘h12' herein) participated in the experiments to examine the presence of tactile sensations associated with focused ultrasound (FUS) sonication, while the rest (*n* = 6, one female, age 28.7 ± 9.0 yrs, range 21–43) underwent measurement of EEG responses to the sonication. Prior to the sonication experiment, magnetic resonance imaging (MRI) and computed tomography (CT) scans were conducted across all the participants for both neuroradiological assessment and for the preparation of the image-guided application of FUS. The presence of clinically-significant calcification that may interfere with and perturb the acoustic propagation within the cranial cavity was examined using the CT data (none were found).

On the day of the sonication experiment, prior to the sonication, neurological examination (NE) was provided to each subject by licensed neurologists. Then, FUS was administered on the hand primary somatosensory cortex of each hemisphere based on real-time image-guidance of the acoustic focus with respect to the subject-specific structural and functional neuroanatomy. A separate ‘sham'-type FUS session, which mimicked the experimental procedures without providing actual sonication, was also given while the sequence was randomized and balanced across the participants. Then, the subject was monitored for 2 h to assess the presence of any short-term neurological effects, whereby a second set NE was performed. All the participants were subsequently divided into four groups and underwent a follow-up anatomical MRI at different time periods after the FUS, *i.e.* during the same day (*n* = 7), 2 weeks (*n* = 3), 4 weeks (*n* = 6), and 8 weeks (*n* = 2). All the participants were re-contacted by telephone two months after the sonication session to be interviewed about the presence of any changes in their mental and physical health status, including experiences of any discomfort.

### Sonication Setup

A ceramic piezoelectric FUS transducer operating at 250 kHz (Channel Industries, Santa Barbara, CA) was used to generate the acoustic pressure wave for the sonication of hand primary somatosensory cortex (S1). The transducer was shaped as a segmented-sphere with an outer diameter of 6 cm and a radius-of-curvature (ROC) of 7 cm, and was housed in an air-backed, water-proof plastic casing. The transducer was immersed in degassed water that was contained in a cone-shaped, thin film bag (linear low-density polyethyelene; LLDPE; approximately 75 μm in thickness). The film material did not introduce any measurable reduction or distortion in the path of the acoustic beam. The transducer was connected to an applicator that was installed on mechanical arms, which allowed the operator to manually adjust and lock the location of the FUS transducer in a specific orientation. Image-guidance was used to help the operator align the acoustic focus on the target location of the subject's cortical structures (schematics of the image-guidance sonication setup are shown in Fig. 1). The hair was carefully combed away from the entry point, and the ultrasound hydrogel (Aquasonic, Parker Laboratories, Fairfield, NJ, USA) was applied between the bag and scalp.

The FUS transducer was actuated using electrical signals that were generated by two signal generators (33220A; Agilent technologies, Inc., Santa Clara, CA) and were subsequently amplified by a class-A power amplifier (Electronics and Innovations, LTD, Rochester, NY). A calibrated needle-type hydrophone (HNR500; Onda, Sunnyvale, CA) was used to characterize the acoustic power output of the transducer by correlating the relationship of the voltage amplitude of the driving electrical signal and acoustic intensities at the focus. The spatial profile of the acoustic focus was measured by the hydrophone mounted on the 3-axis robotic stage (Bi-Slides; Velmex, Bloomfield, NY) and is shown in Fig. 1d. The detailed method of the transducer characterization was described elsewhere^21^. The focal diameter was estimated in the transverse plane perpendicular to the incident sonication beam path (30 × 30 mm^2^ square area, 1 mm step) at the distance of the ROC of the transducer (based on the time-of-flight information), and the length of the focus was measured along the beam path (50 × 150 mm^2^ rectangular area, 1 mm step). The size of the focus was 7 mm in diameter and 47 mm in length along the sonication axis at the full-width at half-maximum (FWHM) of the acoustic intensity map. The centroid coordinates of this focus were represented as the focal point coordinates for later image-guidance.

The sonication session consisted of batches of sonication trials that were 3 s apart (Fig. 1e). Each trial provided a sinusoidal acoustic pressure wave of 250 kHz, operating at a tone-burst-duration of 1 ms with a pulse repetition frequency (PRF) of 500 Hz (*i.e.* duty cycle of 50%) and a sonication duration of 300 ms to elicit excitation in the S1. The acoustic intensity at the FUS focus, without the presence of the skull, had a spatial-peak pulse-average acoustic intensity (I_sppa_) of 3 W/cm^2^, resulting in a spatial-peak temporal-average acoustic intensity (I_spta_) of 1.5 W/cm^2^. A low incident acoustic intensity of 3 W/cm^2^ I_sppa_ was in compliance with the international electrotechnical commission (IEC) 60601 part 2 standard for physiotherapy equipment^55^,^56^. At each acoustic intensity level, a corresponding mechanical index (MI) was calculated to describe the likelihood of biological effects due to cavitation in the tissues^56^.

### Multi-Modal Imaging Acquisition and Processing for Sonication Planning

Prior to the acquisition of the neuroimage data, four sets of donut-shaped, multi-modal, (*i.e.* visible in both MRI and CT scans) self-adhesive fiducial markers (PinPoint; Beekley Corp., Bristol, CT) were placed onto the subject's head. These fiducial markers were later used to co-register the spatial coordinates of the subject-specific neuroimaging data with the actual head anatomy. The center of the fiducial markers was aligned with the subject's natural anatomical locations, such as skin blemishes (dark spots) and bifurcation of the surface veins, to allow for reproducible placement of the fiducial markers between the imaging session and the sonication experiment. These fiducial markers were placed on spatially distributed locations to minimize target registration error (TRE) during the registration process^57^.

The anatomical information on the skull structure was obtained using a clinical CT scanner (Aquilion ONE, Toshiba), which imaged most of the participant's head (axial orientation, slice thickness = 0.5 mm, field-of-view (FOV) = 24 × 24 cm^2^, image matrix = 512 × 512, voxel size = 0.47 × 0.47 × 0.5 mm^3^). MRI was also performed to obtain anatomical as well as functional information of the brain. A 3-Tesla clinical MRI scanner (MAGNETOM Skyra, Siemens) with a 4-channel head coil was used. Anatomical T_1_-weighted images (3D GRAPPA sequence, acceleration factor = 2, TR/TE = 1900/2.46 ms, Flip angle = 9°, slice thickness = 0.94 mm, FOV = 24 × 24 cm^2^, image matrix = 256 × 256, voxel size = 0.94 × 0.94 × 0.94 mm^3^) were acquired in the sagittal orientation covering the entire telencephalic areas of the head. Major arteries in the brain were also imaged using a Magnetic Resonance Angiogram (MRA) sequence (axial orientation, time-of-flight 3D multi-slab GRAPPA sequence, TR/TE = 23/3.98 ms, flip angle = 15°, acceleration factor = 2, slice thickness = 0.6 mm, FOV = 24 × 24 cm^2^, image matrix = 896 × 896, voxel size = 0.27 × 0.27 × 0.6 mm^3^). The MRA information was used to assess the presence of any vessel abnormality, such as arterial aneurysm.

To identify the individual-specific location of the hand somatosensory cortex of the brain, functional MRI (fMRI) was performed using a gradient-echo echo-planar-imaging (EPI) sequence (TR/TE = 2500/30 ms, flip angle = 90°, slice thickness = 4 mm, FOV = 24 × 24 cm^2^, image matrix = 96 × 96, voxel size = 2.5 × 2.5 × 4 mm^3^). The scanning orientation was set obliquely to image the plane parallel to the imaginary plane connecting the anterior commissure (AC) and the posterior commissure (PC) of the brain. To elicit the functional activation of the sensory areas of the hand that accompanies the motor tasks, subjects were asked to clench their right or left hand about twice per second during a task period of 25 s while the visual cue, synchronized with the scanner operation, was delivered to subjects *via* an MRI-compatible screen (E Sys fMRI, Invivo, Gainesville, FL). The three blocks of the task period were interleaved by four resting periods of equal duration while the subjects were asked to lie still. A dummy scan of 8 s was included in the beginning of the imaging session to allow for T_1_ signal equilibration, which was not included in the data processing. The fMRI data was processed by the SPM8 software package (Wellcome Department of Imaging Neuroscience, University College London, London, UK; www.fil.ion.ucl.ac.uk/spm), whereby the task-related neuronal activity was estimated by a general linear model (GLM) after motion correction. The degree of voxel-wise statistic parametric map in *t*-value, with respect to the task-specific canonical hemodynamic response function (HRF), was obtained and stored in the digital imaging and communications in medicine (DICOM) format for the subsequent multi-modal image registration.

The acquired MRI, fMRI, and CT data were co-registered prior to loading them in an image-guided FUS navigation software that was developed in-house^58^. For co-registration, we adopted the Normalized Mutual Information technique^59^ for volumetric registration, whereby T_1_-weighted volumetric magnetic resonance images were used as the registration target (*i.e.* a fixed volume). The quality of spatial registration was evaluated by examining the locations of the fiducial markers that were placed on the skin during both the MRI and CT imaging.

### FUS Navigation and Guidance

A recliner chair was provided to the subjects to promote comfort during the sonication session. Each subject was asked to tilt their head laterally to accommodate the positioning of the transducer applicator and was also asked to stay as still as possible. To register the coordinate system for the subject's head to that of the co-registered multi-modal neuroimaging data, the fiducial markers were placed to the same locations used during the image acquisition. Subsequently, the spatial transformation matrix was calculated by linking the coordinates of fiducial markers to their corresponding image space (by digitization with an optical pointer tool). The fiducial registration error (FRE), which is the root-mean-square (RMS) distance between corresponding fiducial points after registration^57^, was maintained to be less than 5 mm. The registration quality between the subject's head and the corresponding neuroanatomical images was assessed by navigating through several anatomical features on the subject's head (nose tip, forehead, and midline along the head) and the fiducial markers before the application of the sonication. The subject's head motion in relation to the sonication system was tracked in real-time by a goggle-type rigid-body tracker (Fig. 1a, indicated as ‘head-motion tracker' containing four infrared-reflective markers). The subject's head was not restrained by any means, but the subject was discouraged from head movement during the registration session and the FUS sonication session.

A rigid-body tracker that was attached to the FUS transducer provided its spatial coordinates and orientations in real-time, as detected by the optical tracking system (NDI, Ontario, Canada)^58^ (Fig. 1a, indicated as ‘transducer tracker' containing four infrared-reflective markers). The target sonication area was prescribed to the hand S1 (located in postcentral gyrus) of each hemisphere based on the fMRI activation map (local maxima of activation probability) and the anatomical MRI (Figs. 1b and c, designated as the ‘red dot'). The location of the focal point (Figs. 1b and c, shown as the intersection of the two green crosshairs) and the path of the FUS (Fig. 1b, upper panels, the angled green line) with respect to the transducer was displayed and updated on the monitor in real-time. The operator manually adjusted the location and spatial orientation of the transducer to position the FUS focus on the target area. The incident acoustic beam was aligned to be as perpendicular as possible to the skull curvature to minimize the travel distance as well as to reduce the deviant wave propagation through the skull.

### Elicitation of Explicit Tactile Sensations by FUS Stimulation

FUS (consisting of ~200 stimulation trials) was administered on the hand S1 of each hemisphere, one hemisphere at a time, in a randomized sequence. The location of the FUS transducer was maneuvered slightly around the targeted area until the subject informed the operator about the presence of a sensation. When the sensation was elicited by FUS sonication, the subjects signaled the presence of sensation using a touch sensor attached to their non-targeted hand (ipsilateral side to the FUS sonication). Subjects were also instructed to give a short description of the elicited sensations (*i.e.* type of sensation and where it occurred) using their own words. The sonication was given without the presence of any peripheral sensations at the skull (see results section). The spatial information of the sonication setup was recorded when the subject reported the sensation(s) and was used for retrospective numerical simulations of the acoustic profile for a FUS trial with successful elicitation of a sensation.

### Somatosensory Cortical Potential evoked by FUS Stimulation

To quantitatively examine the evoked brain responses from the FUS stimulation, electroencephalography (EEG) recording was performed during the FUS stimulation on the unilateral primary somatosensory cortex from six healthy volunteers (the choice of hemisphere was randomized and balanced). The EEG data were acquired from two cup electrodes (Neuroline; Ambu, Denmark) placed at electrode sites of C3 and P3 using a dual-channel hardware (BioAmp, ML408; ADInstruments, CO) and software (PowerLab; ADInstruments, CO). A reference electrode was applied to the ipsilateral mastoid and a ground electrode was placed on the ipsilateral ulnar styloid process. The data were filtered online with a 60 Hz notch filter and a bandpass filter of 0.3–200 Hz prior to undergoing subsequent off-line analysis.

Before the FUS session, the EEG signals, without providing any external stimuli, were measured 100 times at an interval of 2 s and averaged to confirm that there was no signal contamination from the environment. Subsequently, the somatosensory evoked potential (SEP), in response to a weak electrical stimulations (2 s intervals, averaged from *n* = 100, each 200 μs square-wave pulse, current intensity adjusted according to the individual's comfort level up to 1.2 mA) to the median nerve at the wrist contralateral to the FUS-targeted hemisphere (delivered through a bar electrode, MLA DDF-30, generated by ML408; ADInstruments, CO). Then, the sonication-specific evoked potential (EP) during the image-guided transcranial FUS to the somatosensory cortex was measured in the absence of electrical stimulation. The sham session was also conducted (randomized in sequence in relation to the FUS session). Statistical analyses (paired *t*-test; two-tailed) were performed between the SEP and those of the FUS-evoked EEG responses.

### Subject-Specific Acoustic Simulations

The direct visualization of the acoustic pressure profile in humans *in vivo* is not feasible at the tested level of acoustic intensity due to the use of an extremely low pressure level and radiation force, which cannot be detected by the current state of acoustic radiation force impulse (ARFI) imaging^60^. Moderate heating of the brain tissue and its subsequent detection using MR thermometry, which is often used in high-intensity FUS ablation^20^,^61^, was not applicable to healthy individuals. Therefore, the location and the acoustic intensity of the FUS focus inside of the brain were retrospectively estimated using acoustic simulation software (Wave3000; Cyberlogic, New York, NY). The anatomy of the individual subject's skull (obtained from the CT) and the information of the sonication orientation, which resulted in the elicitation of the stimulation-related response, were used in the simulation. The simulation covered a 30 × 30 mm^2^ square area around the center of the targeted FUS focus (with a 2 mm step) in a transversal plane perpendicular to the sonication path. To visualize the sonication profile along the acoustic beam path, the acoustic simulation was also performed in the longitudinal plane covering a 30 × 50 mm^2^ square area (with a 2 mm step) from 9 mm posterior to 41 mm anterior of the focus along the centroid of the targeted sonication path. For the simulation of the acoustic propagation from the participant who did not report any sensation (‘h5' in stimulation of both hemispheres and ‘h8' in the right hemispheric stimulation; Supplementary Table S3), the simulation was conducted with respect to the initial sonication setting.

The material properties were used as provided by the acoustic simulation software (density = 1850 kg/m^3^, λ = 9306 MPa, μ = 3127 MPa, η = 40 Pa·s, ϕ = 0.1 Pa·s for skull; density = 1000 kg/m^3^, λ = 2241 MPa, μ = 0 MPa, η = 0.001 Pa·s, ϕ = 1.0 × 10^−7^ Pa·s for water; where λ = Lamé's first parameter related to the bulk modulus and shear modulus, μ = Lamé's second parameter or the rigidity modulus, η = the shear or first viscosity, ϕ = the bulk or second viscosity). Additionally, the attenuation due to brain tissue was included in the estimation using the intracranial distance of the specific sonication path and the attenuation factor of the brain tissue^62^, *i.e.* 0.0175 Np/cm. Another set of simulations with all the same parameters, but excluding the skull anatomy, was conducted to assess the transmission rate of the acoustic intensity, including the degree of deviation of the acoustic focus from the intended target.

### Estimation of Thermal Effects from the Sonication

The potential temperature increase *via* the FUS sonication was theoretically estimated using the equation reported in a previous study^63^ (*i.e.*, ΔT = 2αIt/ρ_b_C_p_ = 2 × 0.0175 cm^−1^ × 2.5 W·cm^−2^ × 0.3 s/3.811 J·cm^−3^·°C^−1^; where α = the absorption coefficient^62^, I = the attenuated acoustic intensity at the FUS focal area, t = the pulse duration of sonication, ρ_b_ = the density of brain tissue^64^, and C_p_ = the specific heat of the brain tissue^64^). The estimated temperature rise of 0.007°C (ΔT) was much less than the temperature threshold that can induce any tangible thermal bioeffects.

## Author Contributions

W.L., H.K., Y.J., I.-U.S. and Y.A.C. participated in study design, data acquisition and analysis. S.-S.Y. participated in equipment preparations and study design. All participated in manuscript writing.

## Supplementary Material

Supplementary Information Supplementary Information

## Figures and Tables

**Table 1:** The locations and types of tactile sensations elicited by the FUS sonication (out of 11 responsive individuals from ‘h1' to ‘h12'). (a) The locations and (b) the types of sensations, as well as the corresponding number of subjects who reported the sensations. Detailed information in terms of individual and hemisphere-specific responses can be found in the Supplementary Tables S1 and S2

a			b		
Locations of sensations	Ratio of subjects reported		Types of sensations	Ratio of subjects reported	
Hand/Finger(s)	11/11	100.0%	Tingling	11/11	100.0%
Wrist	2/11	18.2%	SOM	11/11	100.0%
Forearm	5/11	45.5%	Heaviness	6/11	54.5%
Elbow	2/11	18.2%	Numbness	2/11	18.2%
Arm	4/11	36.4%	Feeling of weakelectrical current flow	3/11	27.3%
Armpit	1/11	9.1%	Itching	2/11	18.2%
Buttock	1/11	9.1%	Brushing	1/11	9.1%
Foot	1/11	9.1%	Cooling	1/11	9.1%

**Table 2:** The estimated acoustic intensity, in terms of I_sppa_, at the intended target location (AI_@target_) and its maximum value within the simulated region-of-interest (AI^max^_@ROI_), along with the estimated spatial deviations in targeting (represented as ‘Focus shifting'), based on the acoustic simulation. The skull thickness was also measured along the sonication path. Note that the incident acoustic intensity was 3 W/cm^2^ I_sppa_

I_sppa_ (W/cm^2^)					
ID	Hemi	AI_@target_	AI^max^_@ROI_	Focus shifting (mm)	Skull thk (mm)
h1	L	0.9	1.0	1.0	6.8
	R	0.5	0.5	1.0	8.2
h2	L	0.4	0.8	4.2	6.4
	R	0.7	2.3	9.5	8.7
h3	L	1.1	1.2	1.0	4.7
	R	1.1	1.1	0.0	5.1
h4	L	0.8	1.1	1.0	7.4
	R	1.5	1.5	1.0	5.3
h5	L	0.1	0.8	13.0	10.4
	R	0.2	1.1	11.0	10.5
h6	L	0.5	0.6	1.4	8.1
	R	1.2	1.2	0.0	6.1
h7	L	0.3	1.0	5.0	6.2
	R	0.7	0.8	1.4	5.4
h8	L	0.6	0.7	1.0	6.8
	R	0.5	1.1	9.5	7.8
h9	L	2.5	2.5	1.0	7.6
	R	0.8	1.3	1.0	8.3
h10	L	0.4	1.1	11.0	9.5
	R	0.6	2.3	3.2	9.8
h11	L	0.3	0.7	3.2	9.1
	R	0.7	0.8	1.0	7.4
h12	L	0.6	0.9	1.0	7.6
	R	0.7	0.8	1.0	7.8
	Mean	0.7	1.1	3.5	7.5
	s.d.	0.5	0.5	4.1	1.6
