# Supplementary material for: Image-Guided Transcranial Focused Ultrasound Stimulates Human Primary Somatosensory Cortex
Source: Sci Rep. 2015 Mar 4;5:8743. doi: 10.1038/srep08743 (PMC4348665; doi:10.1038/srep08743)
Supplement: Supplementary Information [file srep08743-s1.doc]

**Supplementary Information**

Image-Guided Transcranial Focused Ultrasound

Stimulates Human Primary Somatosensory Cortex

Wonhye Lee^1,3^, Hyungmin Kim^1,2^, Yujin Jung^1^, In-Uk Song^1^, Yong An Chung^1^, Seung-Schik Yoo^1,3^

^1^Incheon St. Mary’s Hospital, The Catholic University of Korea, Incheon 403-720, Korea

^2^Center for Bionics, Korea Institute of Science and Technology, Seoul 136-791, Korea

^3^School of Nano-Bioscience and Chemical Engineering, Ulsan National Institute of Science and Technology, Ulsan 689-798, Korea

- FUS-mediated Stimulation of Somatosensory Cortex -

**Supplementary Table S1.** The locations of sensations reported from the FUS session for each individual. L: left hemisphere, R: right hemisphere.

|  | h1 | | h2 | | h3 | | h4 | | h5 | | h6 | | h7 | | h8 | | h9 | | h10 | | h11 | | h12 | |
| --- | --- | --- | --- | --- | --- | --- | --- | --- | --- | --- | --- | --- | --- | --- | --- | --- | --- | --- | --- | --- | --- | --- | --- | --- |
| Sensation Locations | L | R | L | R | L | R | L | R | L | R | L | R | L | R | L | R | L | R | L | R | L | R | L | R |
| Hand/Finger(s) | Y | Y | Y | Y | Y | Y | Y | Y | N | N | Y | Y | Y | Y | Y | N | Y | Y | Y | Y | Y | Y | Y | Y |
| Wrist | N | N | N | N | N | N | N | N | N | N | N | N | Y | Y | Y | N | N | N | N | N | N | N | N | N |
| Forearm | N | N | N | Y | N | N | N | N | N | N | N | N | Y | N | Y | N | N | N | N | Y | N | Y | N | N |
| Elbow | N | N | N | Y | N | N | N | N | N | N | N | N | N | Y | N | N | N | N | N | N | N | N | N | N |
| Arm | N | Y | N | N | N | N | N | N | N | N | N | N | N | Y | N | N | N | Y | N | N | N | Y | N | N |
| Armpit | N | N | N | N | N | N | N | N | N | N | N | N | N | N | N | N | N | Y | N | N | N | N | N | N |
| Buttock | N | N | N | N | N | N | N | N | N | N | Y | N | N | N | N | N | N | N | N | N | N | N | N | N |
| Foot | N | N | N | N | N | N | N | N | N | N | Y | N | N | N | N | N | N | N | N | N | N | N | N | N |
|  | Y: Reported, N: Not reported | | | | | | | | | | | | | | | | | | | | | | |  |

**Supplementary Table S2.** The types of sensations reported by each individual from the FUS session. L: left hemisphere, R: right hemisphere.

|  | h1 | | h2 | | h3 | | h4 | | h5 | | h6 | | h7 | | h8 | | h9 | | h10 | | h11 | | h12 | |
| --- | --- | --- | --- | --- | --- | --- | --- | --- | --- | --- | --- | --- | --- | --- | --- | --- | --- | --- | --- | --- | --- | --- | --- | --- |
| Sensation  Types | L | R | L | R | L | R | L | R | L | R | L | R | L | R | L | R | L | R | L | R | L | R | L | R |
| Tingling | Y | Y | Y | Y | Y | Y | Y | Y | N | N | Y | Y | Y | Y | Y | N | Y | N | Y | Y | Y | Y | Y | Y |
| SOM | Y | Y | N | Y | N | Y | Y | N | N | N | N | Y | Y | Y | Y | N | Y | Y | N | Y | Y | Y | Y | N |
| Heaviness | Y | Y | Y | Y | N | N | N | N | N | N | Y | N | Y | N | N | N | Y | Y | N | N | Y | N | N | N |
| Numbness | N | N | Y | N | N | N | N | N | N | N | N | N | N | N | N | N | N | N | Y | Y | N | N | N | N |
| Feeling of weak electrical current flow | N | N | N | N | N | N | N | N | N | N | N | Y | Y | Y | N | N | Y | N | N | N | Y | N | N | N |
| Itching | N | N | N | N | N | N | N | N | N | N | N | N | N | N | N | N | N | Y | N | Y | N | N | N | N |
| Brushing | N | N | N | N | N | N | N | N | N | N | N | N | N | N | Y | N | N | N | N | N | N | N | N | N |
| Cooling | N | N | N | N | N | N | N | N | N | N | N | N | N | N | N | N | N | N | N | N | N | Y | N | N |
|  | Y: Reported, N: Not reported | | | | | | | | | | | | | | | | | | | | | | | |

**Supplementary Table S3.** The number of responsive FUS stimulations across subjects 'h1' to 'h12'. Hemi: hemisphere, L: left, R: right.

| ID | Hemi | Ratio of responsive FUS stimulation | |
| --- | --- | --- | --- |
| h1 | L | 164 / 200 | 82.0% |
|  | R | 102 / 135 | 75.6% |
| h2 | L | 178 / 200 | 89.0% |
|  | R | 177 / 200 | 88.5% |
| h3 | L | 129 / 200 | 64.5% |
|  | R | 105 / 200 | 52.5% |
| h4 | L | 61 / 200 | 30.5% |
|  | R | 145 / 200 | 72.5% |
| h5 | L | 0 / 200 | - |
|  | R | 0 / 200 | - |
| h6 | L | 52 / 200 | 26.0% |
|  | R | 60 / 200 | 30.0% |
| h7 | L | 151 / 200 | 75.5% |
|  | R | 145 / 200 | 72.5% |
| h8 | L | 119 / 200 | 59.5% |
|  | R | 0 / 200 | - |
| h9 | L | 101 / 200 | 50.5% |
|  | R | 72 / 200 | 36.0% |
| h10 | L | 19 / 160 | 11.9% |
|  | R | 31 / 200 | 15.5% |
| h11 | L | 70 / 200 | 36.1% |
|  | R | 92 / 200 | 46.0% |
| h12 | L | 122 / 200 | 61.0% |
|  | R | 133 / 200 | 66.5% |
|  |  | Mean | 54.4% |
|  |  | s.d. | 23.5% |

**Supplementary Table S4.** The estimated acoustic intensity (I_sppa_) at the intended target location (AI_@target_) and its maximum value within the simulated region-of-interest (AI^max^_@ROI_), along with the estimated spatial deviations from the target ('Focus shifting') and the skull thickness in the sonication path. The data were obtained from the subjects ('h13' through 'h18') who participated in the FUS-EEG experimental session.

|  |  | I_sppa_ (W/cm^2^) | | Focus shifting (mm) | Skull thk (mm) |
| --- | --- | --- | --- | --- | --- |
| ID | Hemi | AI_@target_ | AI^max^_@ROI_ |
| h13 | R | 0.5 | 0.8 | 1.0 | 7.2 |
| h14 | R | 1.4 | 1.4 | 0 | 5.4 |
| h15 | R | 0.6 | 0.9 | 3.2 | 9.6 |
| h16 | L | 1.0 | 1.1 | 1 | 6.4 |
| h17 | L | 1.5 | 1.5 | 0 | 4.0 |
| h18 | L | 1.3 | 1.3 | 0 | 5.6 |
|  | Mean | 1.0 | 1.2 | 0.9 | 6.4 |
|  | s.d. | 0.4 | 0.3 | 1.2 | 1.9 |
